# Tubercular Ascites Simulating Ovarian Hyperstimulation Syndrome following In Vitro Fertilization and Embryo Transfer Pregnancy

**DOI:** 10.1155/2013/176487

**Published:** 2013-09-18

**Authors:** Amar Ramachandran, Pratap Kumar, Naveen Manohar, Raviraj Acharya, Anita Eipe, Rajeshwari G. Bhat, Lorraine Simone Dias, Padmaja Raghavan

**Affiliations:** ^1^Division of Reproductive Medicine, Department of OBG, Kasturba Medical College, Manipal University, Manipal, Karnataka 576104, India; ^2^Department of Medicine, Kasturba Medical College, Manipal University, Manipal, Karnataka 576104, India

## Abstract

Ovarian hyperstimulation syndrome (OHSS) is a known complication of using ovulation induction drugs in assisted reproductive techniques. Its incidence and severity vary. Tuberculosis is a very common disease in the developing world, and ascites is one of its sequelae. The newer aids in diagnosing tuberculosis include measuring levels of Adenosine DeAminase (ADA) in the third-space fluids or serum. This case report is from a tertiary care center, reflecting how tubercular ascites simulated OHSS, and the right diagnosis was made and managed. This is being presented due to its rarity.

## 1. Introduction

Ovarian hyperstimulation syndrome (OHSS) is a well-known complication of assisted reproductive techniques (ARTs) and is characterized by enlargement of the ovaries and fluid shift from the intravascular compartment to the third space [1]. Tuberculosis is common in developing countries, and peritoneal tuberculosis (TB) which is the 6th most frequent extrapulmonary TB usually presents with ascites. We report a case of a 31-year-old lady who presented with tubercular ascites that simulated ovarian hyperstimulation (OHSS). The patient had no evidence of tuberculosis as proven by a negative Mantoux, chest X-ray, acid fast staining of ascitic fluid, and a negative PCR. The final diagnosis and management were based on a rising Adenosine DeAminase (ADA) level and a low haematocrit of 19.8%. The uniqueness lies in the yet unreported simulation leading to a suspicion of an unknown pathological mechanism in stimulating the ovaries, which might have caused a flare-up of tuberculosis.

## 2. Case Report

A 31-year-old lady came to us with evidence of spontaneous abortion at 14 weeks of her pregnancy, which was conceived following in vitro fertilization. An ultrasound scan done showed an empty uterine cavity, indicating a complete abortion. She had fever at the time, and hence a course of antibiotics was given. Her hemoglobin levels were low, for which she was given a unit of packed red blood cells.

She was a booked case with us and had a past history of two episodes of ascites (OHSS) following the embryo transfer. The first episode was within 12 days of embryo transfer, and the second episode was at 9-10 weeks of gestation. Both episodes were diagnosed as OHSS and treated symptomatically with albumin infusion. At 14 weeks of gestation, she had fever and recurrence of ascites. Ascites did not subside even with albumin and Cabergoline; hence other causes of ascites were evaluated by Mantoux test and chest X-ray, which were negative for tuberculosis.

Her bleeding per vaginum persisted, for which a scan was done again which showed some retained products of conception for which she underwent dilatation and evacuation. The tissue obtained was sent for histopathology examination and came back as only degenerated products of conception and negative for mycobacterium tuberculosis by PCR.

When the ascites did not disappear after the regular treatment, the ascitic fluid was tapped thrice. It was green in color, leading to suspicion of presence of bile salts or pigments in it though her liver function tests were normal. When analyzed for the same, there were no bile salts or pigments found. Upon culturing it, no growth was found after 7 days. The ascitic fluid was negative for malignancy, and Ca 125 was normal. Her hematocrit persisted at the same values (19.8%) as before. She had lost significant weight within two weeks. The ascitic fluid was further investigated with acid fast staining, which showed no acid fast bacilli. A PCR sent for mycobacterium tuberculosis came back negative. Further evaluation showed its Adenosine DeAminase (ADA) level was 78 IU/L and rose to 110 IU/L in 3 days (normal: <39 IU/L). Based on the ADA levels, she was started on antitubercular treatment with HRZE (Isoniazid + Rifampicin + Pyrazinamide + Ethambutol), which finally resolved the ascites within a week.

## 3. Discussion

 Ovarian hyperstimulation is a rare complication of using GnRH antagonist protocol for ovulation induction in patients undergoing assisted reproductive techniques [2]. When mild, it only presents with pelvic or abdominal discomfort. But when it gets severe, like it rarely does, shortness of breath and orthopnoea due to pleural effusion are also seen. Upon using ovulation induction drugs, the ovaries are being constantly pushed with respect to their state of function. But what really causes them to go into overdrive and start secreting excessive fluid is not well understood yet. One of the most commonly seen risk factors is polycystic ovaries [3].

Pathologically, in ovarian hyperstimulation, the fluid lost is “transudate” in nature. Clinically, it is seen as a sudden increase in the weight of the patient along with some pelvic pains. Usually, it is a mild complication, which does not require much intervention. But in some cases, it can cause massive ascites and proceed to a pleural effusion leading to life-threatening dyspnoea. Furthermore, investigating with an ultrasound will show fluid in the peritoneal cavity which might also be in the pleural cavity, which can be confirmed with a chest X-ray. The routine treatment involves a 100 mL of saline with 20% albumin infusion intravenously, which will help bring the excessive fluid back into the intravascular compartment [4]. Another effective drug is Cabergoline, which acts by influencing the vasoactive endothelial growth factor (VEGF) pathway, restricting the loss of fluid into third spaces [5].

Similar presentation is seen with ascites due to tuberculosis of the peritoneum. Tuberculosis is very commonly seen in the developing countries and usually affects the lungs primarily. Any other organ involved is secondary. So, a clear chest X-ray is usually taken to mean no tuberculosis anywhere else. When it affects the peritoneum and produces ascites, it is an “exudate”.

The difference in the two underlying pathologies of ascites is that when a transudate is seen, concentration of hematocrit can be seen [6]. On the other hand, in exudative ascites, there is no hemoconcentration seen. It was the persisting hematocrit at low levels (19.8%) even with ongoing ascites that led to a suspicion of a cause other than hyperstimulation of the ovaries.

Tuberculosis is, commonly, a disease of the developing countries. Most of the population in such a country is affected, but a good immunity keeps the disease in check, not letting it flare up. But whenever a body experiences a kind of stress, it loses part of its immunity, and this, in turn, leads to flaring up of tuberculosis. Some of the stresses known to cause flaring up are immunosuppression therapy, nephropathy, and so forth. This case gives rise to the possibility that ovulation induction might actually be a stress factor in causing flaring up of tuberculosis. There is not enough evidence to test the theory yet [7].

Estimation of Adenosine DeAminase (ADA) in ascetic fluid has value in diagnosing tuberculosis. ADA is a purine-degrading enzyme, widely distributed in tissues and body fluids, necessary for proliferation and differentiation of T lymphocytes. The optimal cutoff value is 39 IU/L. It has enough discriminatory power to either confirm or rule out the diagnosis of peritoneal TB. It can be used as a reliable test to start treatment while waiting for the report of cultures. A meta-analysis of 4 studies showed a sensitivity of 99 to 100% and specificity of 97% in diagnosing peritoneal TB [8].

In our case though PCR and acid fast staining were negative for tuberculosis, the persisting low haematocrit levels along with rising ADA levels led to the suspicion of tubercular ascites, and when treated with ATT, ascites was resolved completely.
